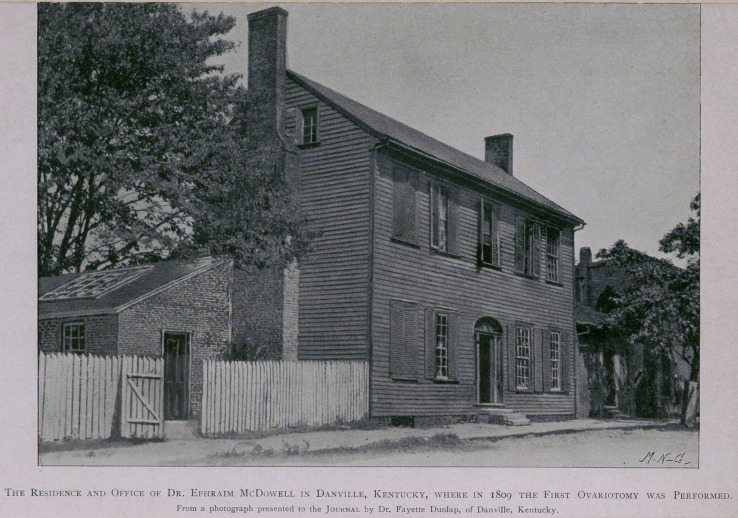# Internal Œsophagotomy

**Published:** 1890-05

**Authors:** John O. Roe

**Affiliations:** Rochester, N. Y., Chairman Section of Laryngology and Otology, American Medical Association; Fellow of the American Laryngological Association, etc.; 28 North Clinton Street


Buffalo Medical and Surgical Journal

Vol. XXIX. MAY, 1890. No. 10.

### Original Communications.

### INTERNAL ŒSOPHAGOTOMY.

### THE REPORT OF A CASE.

By JOHN O. ROE, M. D., Rochester, N. Y.,

Chairman Section of Laryngology and Otology, American Medical Association ; Fellow of the American Laryngological Association, etc.

The operation of internal œsophagotomy is so rarely performed that any details concerning it cannot fail to be of interest. Previous to the case now reported, there had been but twenty cases recorded, two of which were performed by the writer of this article. In these two previous cases mentioned,[1] as well as in the one now reported, the operation was performed for the relief of aggravated fibrous or cicatricial stricture of the œsophagus, and was in each case attended with complete success.

An earlier report of the present case was read before the American Laryngological Association, in September, 1888.[2] The present report contains not only the details of the case as presented at that time, but also the further progress of the case, and the present condition of the patient, which gives an enhanced value to the record of this operation.

Mrs. M. L. O., forty-eight years of age, was referred to me, June 3d, 1887, by Dr. Pardee, of Fulton, N. Y., on account of a serious obstruction to deglutition from which she had suffered for fourteen years.

The onset of the dysphagia was very sudden. One afternoon, while eating a chicken sandwich, a piece of the chicken lodged in her throat and caused her to choke quite violently. The chicken she believed to be entirely free from bones, but such could hardly have been the case, for it would seem scarcely possible for a piece of chicken, free from bone or cartilage, to injure the œsophagus sufficiently, or to lodge in it long enough, to excite inflammation or ulceration to the extent necessary to cause the membranous or cicatricial formation presently to be described.

1. The Medical Record, New York, 1882. Vol. XXII., p. 536.
2. Transactions of the American Laryngological Association, 1888, p. 46.

As contributing to the history of this case, I would say that the patient told me of a fall that she had had about six months before this choking occurred. She was thrown from a sleigh upon a sharp ridge of ice, striking on the back of her neck. She believed that she came very near dislocating her neck, as it was very painful and lame for several weeks; and, for a year afterward, she could not turn her head to look behind her, or to look up, without putting her hand to the back of her head to support it. Whatever influence may be attributed to the fall in developing an abnormal condition in the œsophagus, it is still clear that the acute trouble exhibited itself only as a result of the lodgement of the foreign substance just mentioned in the upper part of the œsophagus.

After the choking above described had subsided, the patient found she was unable to swallow any solid substance whatever, and this difficulty continued until the time I saw her. She was, however, able to swallow liquids and semi-solids very slowly, but a solid substance as large as a tomato seed would lodge in her throat and cause her much discomfort until it was expelled. In such a case she would often be obliged to nauseate herself, and sometimes to produce vomiting by running her finger down her throat.

The nutriment which she was able to take consisted of milk, soups, broths, and the like. She could, also, take bread, cake, potatoes and such substances as could be softened and thoroughly incorporated in milk or broth. Everything, however, excepting clear milk, she insisted on being strained, lest it might contain some solid particle that would lodge in her throat. On taking the nourishment, she would place the substance in the back part of her mouth, throw her head slightly back and allow it to run down very slowly. Efforts at swallowing would afford but little assistance.

In this manner she had been able, during these fourteen years, to sustain herself fairly well. The obstruction was, however, slowly increasing and she was, at the same time, losing flesh and strength. Swallowing had now become so difficult that it occupied all her time during the day to get down sufficient nutriment to appease her hunger. She is a tall woman, five feet five and a half inches in height, and has always been rather slender. Her weight, previous to the commencement of this trouble, was 135 pounds; but it was, at the time I first saw her, reduced to 108 pounds.

On attempting to explore her œsophagus with an ordinary small, conical pointed œsophageal bougie, 4 mm. in diameter, the bougie was arrested two and a half inches below the entrance to the œsophagus. I then tried to pass a fine whalebone filiform bougie, 1 mm. in diameter, but also without success. I then employed a small metal olive-shaped bougie, 2 mm. in diameter, mounted on a small copper stem. With this flexible stem, bent in various directions, I finally succeeded in finding the opening. It was located at the extreme left side of the œsophagus, so that the bougie, when in the passage, lay close against the left posterior pillar of the fauces. This success in finding the opening was very gratifying not only to the patient but to myself as well, for the reason that so many attempts to find the passage had been made by different surgeons without success. Her œsophagus was very sensitive to the introduction of instruments, so much so that much irritation and some pain followed the first exploration. This irritation, however, subsided in a short time with the aid of a little morphine, locally applied; and on the second day afterward, the same bougie was introduced with little or no irritation. This was followed up by slightly larger bougies, until one 6 mm. in diameter could be passed.

For a short time after each dilatation deglutition was improved; but the stricture would soon contract to its former size. Daily dilatation was, nevertheless, persevered in for three weeks, without any additional gain. I then decided to divide the stricture by internal incision, as the easiest and best way of dealing with it. With a bulbous bougie, I determined the vertical extent of the stricture to be about 3 mm. Below this point the œsophagus was entirely free.

By means of the half-round or "demi-bulbous" bougie, I determined that the stricture occupied about two-thirds of the circumference of the œsophagus on the right side. This is the form of bougie I used in my other cases. It was devised by Trelat[1] in 1870. It is of the utmost value in determining the depth of the projecting portion of the stricture, and the point where the incision should be made. The bulb of this bougie is made flat on one side and half-round on the other, forming a half sphere.

It is readily seen that when the bulb is introduced beyond the stricture and withdrawn, if the bulbous side is turned towards the projecting portion of the stricture, it will meet with resistance proportionate to the size of the bulb and to the distance that the edge of the stricture projects beyond the line of the wall of the œsophagus. If the bulb is turned to the flat or straight side of the œsophageal wall, and the flat side of the bulb to the strictured side, no resistance is felt on withdrawing the bulb. If the stricture projects equally on all sides, the same resistance will accordingly be felt when the bulb is turned to either side. After thus carefully determining the location and extent of the stricture and the depth I wished to make the incision, I introduced the œsophagotome and made an incision 2 mm. deep through the most projecting portion of the stricture on the right side. The blade met with a little resistance, indicating that the tissue composing the stricture was moderately firm and dense. The operation caused but little pain, mainly of a stinging sensation, and there was but little

1. *Bulletin Général de Therapeutique Med. et Chirurg.*, Paris, 1870, tome lxxviii., p. 262.

hemorrhage, three or four expectorations only being mixed with blood. During the following twenty-four hours the patient was permitted to take only a small quantity of milk. There was no pronounced pain or inflammation; and on the second day dilatation was begun to prevent the re-union of the cut surfaces. A bougie 10 mm. in diameter could now be passed quite easily and deglutition was much improved, although no solids could yet be taken.

In one week (July 2d) I repeated the incision, dilatation having failed to increase the opening beyond the 10 mm. At this operation the incision was made back of the former one, the blade being turned backward about 35°. This incision was made 1½ mm. in depth. It caused scarcely any pain, and only a few drops of blood were seen. The treatment after this incision was the same as that before described. A bougie could now be passed 12 mm. in diameter, and deglutition was correspondingly improved. Dilatation was now continued for ten days, with the result of increasing the opening to admit a bougie 13 mm. in diameter.

On July 12th another incision was made like the last one, but on the opposite side of the stricture. A bougie 15 mm. in diameter could now be admitted. Dilatation was continued and on July 19th, one week later, an incision 2 mm. deep was made in the center, between the first and second incisions, and, on July 26th, a fifth incision 2 mm. deep was made between the first and third incision. The last two operations were about the same in every particular as the first one.

After the last incision, a bougie 20 mm. in diameter was passed through quite easily, and the patient was able to take all the solid food she desired without difficulty. The caliber of the œsophagus at the place where the stricture had been was so near its normal size, that further operations were deemed unnecessary. Dilatation was, however, continued daily to prevent any re-contraction during the process of healing.

At the end of three weeks, there was no narrowing of the tube, and the patient went home for four weeks. She was then to return for examination. During this interval, she herself continued daily the dilatation of the stricture with a bougie which I had taught her to introduce. When she returned, I found that a very slight contraction had taken place, but only sufficient to cause the bougie to go through very snugly.

This contraction was, however, overcome by using a bougie 1½ mm. larger. Some force was required to pass this one at first, but the contraction soon yielded.

The form of bougie which I employed in this case, and which I taught the patient to introduce for herself, was made of hard rubber, two and a half inches long, tapering at both ends alike, the middle portion cylindrical, and mounted on a whalebone stem. The distal end of this bougie was tipped with a flexible gum-elastic and conical-pointed bougie, to facilitate the passage of the hard-rubber portion through the fauces, and to direct it into the œsophagus.

This form of bougie I had devised a year before (in 1886) for the dilatation of strictures at the lower end of the œsophagus in a patient brought to me by Dr. F. D. Vanderhoof, of Phelps, N. Y. In this case, however, it soon became apparent that the stricture was caused by malignant disease, hence internal œsophagotomy was not attempted.

The addition of this flexible conical tip to the hard-rubber bougie is of special advantage in many cases of œsophageal stricture. The small flexible point very readily directs the bougie into the stricture, and the long acute angle of the tip prevents irritation and the necessity of using as much force as would be necessary to carry the dilatation to the same extent with the ordinary short, blunt, olive-shaped bougie.

The advantage of this bougie with a whalebone stem over the long gum-elastic bougie is that the hard rubber is far better for dilating, and the slender whalebone stem readily allows the retention of the bougie for a time sufficient to get the benefit of a longer continued pressure, which the long gum-elastic bougie will not readily permit. It is also very much superior to the other form for dilating strictures low down in the œsophagus.

This flexible tipped hard-rubber bougie, like all other forms of bougies, is made in different sizes, to meet the requirements of different cases.

Since devising this form of bougie, I have employed it in preference to all others.[1]

The œsophagotome employed in this case, and also in my other two cases, is the form devised by Sir Morell Mackenzie, except that I have slightly modified it so that the blade can be more accurately adjusted, and the distance to which the blade will be thrown out at each introduction can be more accurately determined. This instrument has done for me most excellent service, and given results which leave nothing to be desired.

The result of this operation will be evident from a brief description of the condition of the patient subsequent to its performance.

1. For a more extended description of this bougie, and an illustration as it is now made, *vide New York Medical Journal*, October 26, 1889. Vol. L., p. 474.

Her general health began immediately to improve, and she recovered her normal weight of 135 pounds, and has since gained five additional pounds.

The dilatation of the œsophagus has remained permanent, and though she was instructed how to use the bougie in case of a relapse, she has not used the instrument but twice in a year and a half. Her throat may be reported as having regained almost its normal condition. No difficulty is experienced in swallowing her food, except, perhaps, in the case of large pieces of meat which have not been properly masticated.

The history of this case shows not only the advisability, but also the necessity, of this operation in those forms of stricture of the œsophagus in which the stricture is composed of more or less elastic tissues, and where it resists all ordinary forms of dilatation.

28 North Clinton Street.

## Figures and Tables

**Figure f1:**